# A dynamic nomogram for predicting axillary pathological complete response and its prognostic value in HER2-positive breast cancer

**DOI:** 10.3389/fonc.2026.1754705

**Published:** 2026-03-09

**Authors:** Xiaona Liu, Xiaoqian Li, Pan Liu, Zeyu Xia, Yujie Xiao, Yiyang Wang, Qianwen Fang, Huimin Zhang

**Affiliations:** 1 Department of Breast Surgery, The First Affiliated Hospital of Xi’an Jiaotong University, Xi’an, China; 2 Faculty of Medicine, Xi’an Jiaotong University, Clinical Medicine, Xi’an, China

**Keywords:** axillary pathological complete response, HER2-positive breast cancer, neoadjuvant therapy, predictive model, surgery de-escalation

## Abstract

**Background:**

Accurate preoperative prediction of axillary pathological complete response (apCR) following neoadjuvant therapy (NAT) is crucial for de-escalating axillary surgery in breast cancer (BC) patients. This study aimed to develop and validate a dedicated preoperative nomogram for predicting apCR in human epidermal growth factor receptor 2 (HER2)-positive BC using readily available clinical variables and to further evaluate the prognostic significance of apCR for survival outcomes within our cohort.

**Methods:**

This retrospective study enrolled 189 patients with initially node-positive, HER2-positive BC who received NAT and anti-HER2 targeted therapy. Predictors were selected via least absolute shrinkage and selection operator (LASSO) regression, and a multivariable logistic regression model was constructed and presented as a nomogram. Model performance was assessed by the area under the receiver operating characteristic curve (AUC), calibration curves, decision curve analysis (DCA), and clinical impact curve (CIC), with internal validation conducted through 1000 bootstrap resamples. The prognostic value of apCR was evaluated using Kaplan-Meier survival analysis and Cox regression.

**Results:**

In total, 115 patients (60.85%) achieved apCR in our cohort. Multivariable analysis identified three independent preoperative predictors of apCR: larger baseline axillary burden (>20 mm; OR = 0.38, 95% CI: 0.19-0.75; P = 0.005), early ultrasonographic response (complete response [CR]: OR = 4.36, 95% CI: 1.46-16.20; P = 0.014; major response [MR]: OR = 3.84, 95% CI: 1.39-12.08; P = 0.014), and receipt of intensive anti-HER2 therapy (OR = 2.03, 95% CI: 1.05-4.00; P = 0.038). The nomogram incorporating these factors demonstrated good discrimination, with an AUC of 0.735 (95% CI: 0.663-0.807), and showed good calibration and clinical utility. Furthermore, patients achieving apCR had significantly superior disease-free survival (DFS) (HR = 0.29, 95% CI: 0.09-0.92, P = 0.036).

**Conclusions:**

Our study developed and validated a preoperative nomogram that predicts apCR in HER2-positive BC by integrating three readily available clinical variables. This model demonstrates the potential to preoperatively identify candidates who may be suitable for axillary de-escalation strategies, pending future multi-center prospective validation. The established prognostic value of apCR in our cohort underscores its relevance as a critical clinical endpoint.

## Introduction

Breast cancer (BC) is the most commonly diagnosed cancer among women and a leading cause of cancer-related deaths (1). Neoadjuvant therapy (NAT) has become the standard regimen for locally advanced BC, particularly human epidermal growth factor receptor 2 (HER2)-positive and triple-negative subtypes (2). The primary objectives of NAT include tumor downstaging to facilitate breast-conserving surgery and serving as an *in vivo* sensitivity test to guide subsequent treatment regimens. Pathological complete response (pCR), and in particular axillary pathological complete response (apCR), has been demonstrated as a reliable surrogate marker for improved long-term survival (3, 4).

The remarkable efficacy of anti-HER2 targeted therapy has substantially increased the rate of apCR among patients initially presenting with node-positive disease (5). This breakthrough has transformed the approach to axillary surgery, generating considerable interest in the clinical community. In patients who achieve apCR, omitting axillary lymph node dissection (ALND) becomes a viable option, thereby reducing the risk of complications such as lymphedema (6, 7). As a result, sentinel lymph node biopsy (SLNB) has emerged as a minimally invasive alternative to ALND in this context. However, the accuracy of SLNB following NAT is limited by high false-negative rates (FNR) (8). To overcome this limitation, targeted axillary dissection (TAD) has been developed (9). This procedure combines SLNB with selective excision of the clip-marked, biopsy-proven metastatic lymph node, and has been demonstrated to achieve acceptably low FNR. Despite these advancements, a crucial preoperative tool remains unavailable. Both SLNB and TAD are intraoperative staging procedures whose value is only realized after NAT has been completed and surgery has begun. Consequently, the accurate preoperative identification of patients who have attained apCR, which would enable surgical planning to be tailored in advance, remains a major clinical challenge. Conventional imaging modalities lack sufficient sensitivity for detecting residual nodal disease, and definitive pathological assessment, although the gold standard, can only be performed retrospectively on surgical specimens (10).

While a number of predictive models for axillary response have been developed, many rely on postoperative parameters such as breast pathological complete response (bpCR) status, thereby limiting their applicability in preoperative decision-making (11, 12). Moreover, a considerable proportion of existing models are derived from heterogeneous cohorts that include all BC subtypes, without adequately accounting for the distinct tumor biology and marked sensitivity to targeted therapies characteristic of HER2-positive disease (13, 14). As a result, there remains a clinical need for a reliable, subtype-specific tool that can preoperatively identify patients who are optimal candidates for axillary de-escalation strategies.

This study aimed to develop and validate a dedicated preoperative nomogram for predicting apCR specifically in HER2-positive BC, utilizing only readily accessible clinical variables. The prognostic significance of apCR was also corroborated within our cohort, thereby offering a comprehensive evidence base for its role as a key endpoint in guiding personalized surgical management.

## Methods

### Patients

**Figure 1:**
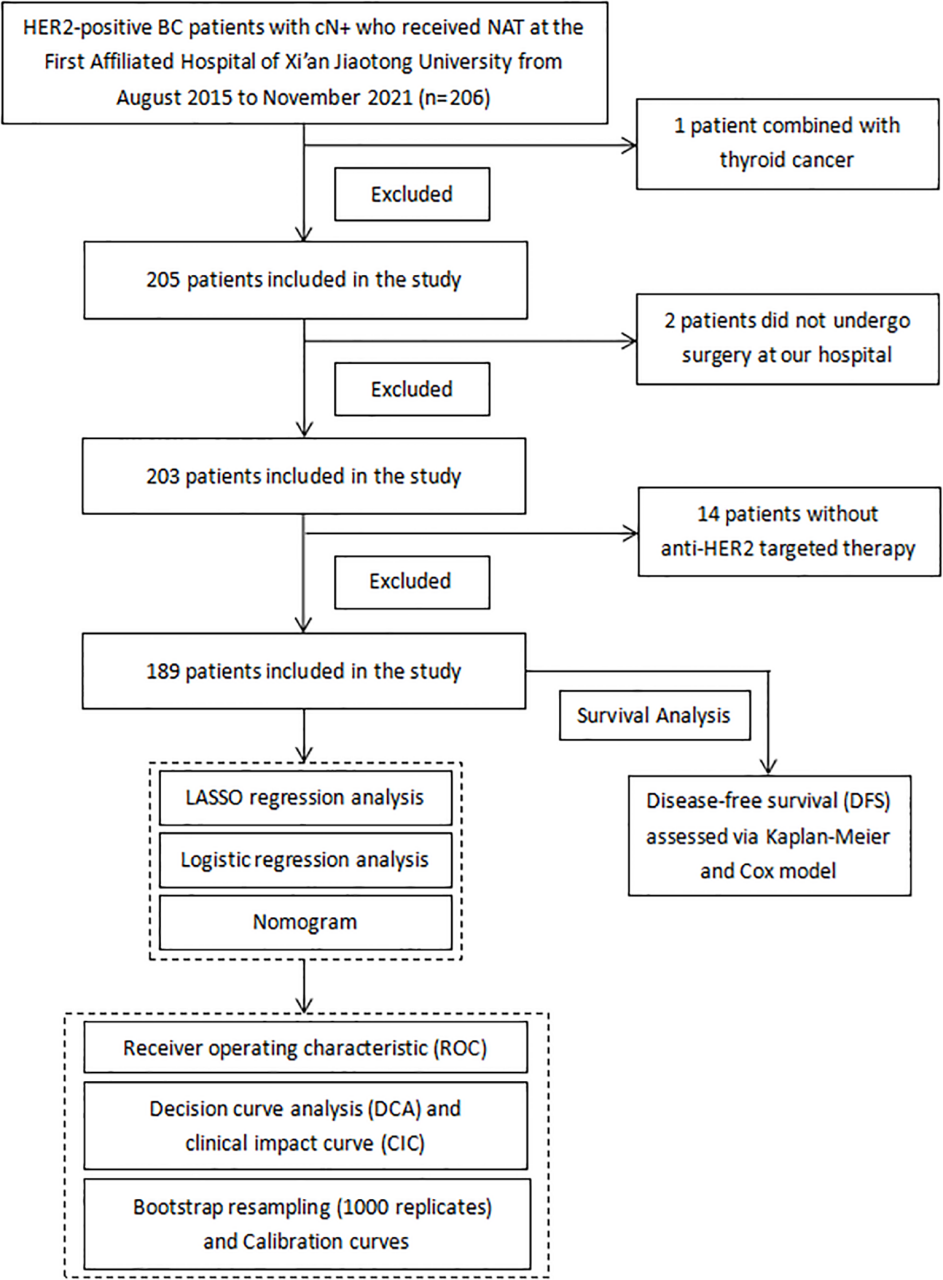
The flowchart of this study.

In our study, we retrospectively screened HER2-positive BC patients who received NAT at the First Affiliated Hospital of Xi’an Jiaotong University from August 2015 to November 2021. The inclusion criteria were as follows: (1) Aged ≥18 years with HER2-positive BC who received NAT. (2) Pathologically confirmed axillary lymph node metastasis prior to NAT via ultrasound-guided core needle biopsy. (3) Comprehensive clinical imaging evaluations confirmed the diagnosis of stage I-III BC. (4) Received at least one anti-HER2 targeted therapy. The exclusion criteria were as follows: (1) Patients with bilateral or occult BC. (2) Patients combined with secondary primary cancer. (3) Patients who completed the NAT but did not undergo surgery at our hospital. (4) Incomplete laboratory or clinical data. Following the application of inclusion and exclusion criteria, a total of 189 patients were included in the final analysis. The study flowchart is presented in **Figure 1**.

### Data collection and efficacy assessments

We collected the following information from the electronic medical records of all patients: clinicopathological data, treatment characteristics, and early ultrasonographic response. The clinicopathological variables included age, menopausal status, hormone receptor (HR) status, HER2 status, Ki-67 index, clinical tumor (cT) stage, clinical lymph node (cN) stage, clinical stage, baseline axillary burden, bpCR, and apCR. HR status was considered positive if ≥1% of tumor cells showed expression of estrogen receptor (ER) and/or progesterone receptor (PR). HER2 expression was considered positive if the immunohistochemistry (IHC) result was 3+ or if the HER2 was confirmed amplified by *in situ* hybridization when the IHC staining was 2+ (15). HER2-positive BC was defined as HER2-positive expression, regardless of HR status. Clinical stage followed the AJCC 8th edition (16). Baseline axillary burden was quantified by the maximum long-axis diameter of axillary lymph nodes measured via ultrasonography prior to NAT initiation. For the 11 cases (5.8%) with missing pretreatment axillary lymph node size, missing values were imputed using the median from the available cohort data. This approach was chosen as a simple, conservative method given the low proportion of missing data and the approximately normal distribution of the variable. The primary endpoint of this study was apCR, defined as the absence of invasive carcinoma in all resected axillary lymph nodes (ypN0) regardless of the response in the breast. bpCR was separately defined as the absence of invasive carcinoma in the breast (ypT0/Tis), regardless of ductal carcinoma *in situ* (DCIS) (17). pCR was assessed by reviewing the final pathological reports of all patients. This assessment was conducted by two independent, experienced pathologists who examined all surgical specimens, with any discrepancies resolved through a consensus review.

Treatment characteristics included the NAT regimen and number of NAT cycles. All patients received standard neoadjuvant chemotherapy, with or without anthracyclines; given the comparable efficacy of these backbones in HER2-positive disease, the regimen was not analyzed as a separate variable (18). Anti-HER2 therapy was categorized into two groups: single-target and dual-target therapy. The dual-target group, defined as the intensive anti-HER2 therapy cohort, comprised patients receiving trastuzumab plus pertuzumab. The single-target group received trastuzumab-based therapy alone.

Early ultrasonographic response was assessed according to Response Evaluation Criteria in Solid Tumors (RECIST) version 1.1 principles (19), with a defined adaptation for axillary lymph nodes. Specifically, the percentage change in the maximum long-axis diameter (as opposed to the short-axis diameter used for target lesions in RECIST) after two cycles of NAT was calculated and categorized as follows: (a) Complete Response (CR): Disappearance of the previously documented lymph node metastasis; (b) Major Response (MR): Reduction ≥50%; (c) Stable Disease (SD): Reduction <50% or increase <30%; (d) Progressive Disease (PD): Increase ≥30%; (e) Missing: Unavailable ultrasonographic measurements. In accordance with a standardized institutional imaging protocol, early ultrasonographic response was assessed by a dedicated breast radiologist with over five years of experience, who was blinded to the patients’ treatment information.

### Model development and validation

Given the single-center nature and moderate sample size of our cohort, we employed robust internal validation techniques rather than data splitting to maximize statistical power and ensure model stability. Least absolute shrinkage and selection operator (LASSO) regression with 10-fold cross-validation was applied to select the most relevant features, and multivariable logistic regression was used to construct the predictive model on the basis of the selected features (20, 21). The events-per-variable ratio for the final multivariable model was approximately 38, indicating a low risk of overfitting. A nomogram was created to provide a visual representation of the model for predicting treatment response. Internal validation was performed with 1000 bootstrap resamples. Discrimination was assessed using the area under the receiver operating characteristic (ROC) curve (AUC) (22). The calibration curve was used to evaluate the agreement between the predicted and observed probabilities. Decision curve analysis (DCA) and clinical impact curve (CIC) were further employed to evaluate the clinical utility of the model (23).

### Survival analysis

We assessed the prognostic value of apCR using disease-free survival (DFS) as the primary endpoint. DFS was defined as the time from surgery to the first event of locoregional recurrence, distant metastasis, contralateral BC, death from any cause, or last follow-up. Patients without events were censored at their last contact date, with a follow-up cutoff of July 2023. For patients lost to follow-up, survival time was conservatively censored at an early truncation date (July 2022) to provide a realistic and unbiased estimate of survival probability. This approach minimizes potential overestimation of survival outcomes that could occur if we assumed all lost patients remained event-free until the end of the study period. The association between apCR status and DFS was evaluated using Kaplan-Meier curves with log-rank testing (24). A univariable Cox proportional hazards model was used to quantify this association, expressed as hazard ratio (HR) with 95% confidence interval (CI).

### Statistical analysis

Continuous variables were described as median (interquartile ranges, IQRs), and categorical variables were summarized as numbers (percentages). Group comparisons were performed using the Mann-Whitney U test or Student’s t test for continuous variables, and the Chi-square test or Fisher’s exact test for categorical variables, as appropriate. All tests were two-sided, and a p value of < 0.05 was considered statistically significant. Statistical analyses were conducted in the R programming environment (version 4.4.2; http://www.r-project.org/). Key analyses were performed using the following R packages: glmnet for LASSO regression, rms for nomogram development and validation, and survival for survival analysis.

## Results

### Patient characteristics

As shown in **Table 1**, a total of 189 eligible patients were included in this study, the overall rate of apCR was 60.85% (115/189). Compared to the non-apCR group, the apCR group had earlier cN stage, earlier overall clinical stage, and significantly smaller baseline axillary tumor burden. Treatment response and therapeutic intensity also differed substantially between groups. Patients achieving apCR showed significantly better early ultrasonographic response to neoadjuvant therapy (P < 0.001), with higher rates of CR (20.00% vs. 5.41%) and MR (16.52% vs. 8.11%), and a markedly lower rate of PD (0.87% vs. 10.81%). Furthermore, the apCR group more frequently received intensive anti-HER2 therapy (59.13% vs. 33.78%, P < 0.001) and completed ≥6 cycles of NAT. A strong association was also observed between bpCR and apCR (73.04% vs. 29.73%, P < 0.001). No significant differences were observed in age, menopausal status, HR status, Ki-67 index, or cT stage.

**Table 1:** Comparison of patient characteristics between the apCR and non-apCR groups.

Variables	All patients N = 189	apCR group N = 115	Non-apCR group N = 74	P value
**Age (years)**	50.00 (43.00-57.00)	50.00 (43.00-57.00)	52.00 (44.25-56.75)	0.625
Menopause, n (%)				0.953
No	95 (50.26%)	58 (50.43%)	37 (50.00%)	
Yes	94 (49.74%)	57 (49.57%)	37 (50.00%)	
HR status, n (%)				0.870
Negative	78 (41.27%)	48 (41.74%)	30 (40.54%)	
Positive	111 (58.73%)	67 (58.26%)	44 (59.46%)	
**Ki-67 index (%)**	40.00 (30.00-60.00)	40.00 (30.00-60.00)	42.50 (30.00-60.00)	0.324
cT stage, n (%)				0.246
cT1	38 (20.11%)	27 (23.48%)	11 (14.86%)	
cT2	120 (63.49%)	72 (62.61%)	48 (64.86%)	
≥cT3	31 (16.40%)	16 (13.91%)	15 (20.27%)	
cN stage, n (%)				0.022
cN1	164 (86.77%)	105 (91.30%)	59 (79.73%)	
≥cN2	25 (13.23%)	10 (8.70%)	15 (20.27%)	
Clinical stage (AJCC 8th), n (%)				0.018
Stage II	138 (73.02%)	91 (79.13%)	47 (63.51%)	
Stage III	51 (26.98%)	24 (20.87%)	27 (36.49%)	
Baseline axillary burden, n (%)				0.006
≤20mm	122 (64.55%)	83 (72.17%)	39 (52.70%)	
>20mm	67 (35.45%)	32 (27.83%)	35 (47.30%)	
Early ultrasonographic response, n (%)				<0.001
SD	88 (46.56%)	45 (39.13%)	43 (58.11%)	
CR	27 (14.29%)	23 (20.00%)	4 (5.41%)	
MR	25 (13.23%)	19 (16.52%)	6 (8.11%)	
PD	9 (4.76%)	1 (0.87%)	8 (10.81%)	
Missing	40 (21.16%)	27 (23.48%)	13 (17.57%)	
Intensive anti-HER2 therapy, n (%)				<0.001
No	96 (50.79%)	47 (40.87%)	49 (66.22%)	
Yes	93 (49.21%)	68 (59.13%)	25 (33.78%)	
NAT cycles, n (%)				0.042
<6	35 (18.52%)	16 (13.91%)	19 (25.68%)	
≥6	154 (81.48%)	99 (86.09%)	55 (74.32%)	
bpCR, n (%)				<0.001
No	83 (43.92%)	31 (26.96%)	52 (70.27%)	
Yes	106 (56.08%)	84 (73.04%)	22 (29.73%)	

apCR, axillary pathological complete response; HR, hormone receptor; cT, clinical tumor; cN, clinical lymph node; SD, Stable Disease; CR, Complete Response; MR, Major Response; PD, Progressive Disease; HER2, human epidermal growth factor receptor 2; NAT, neoadjuvant therapy; bpCR, breast pathological complete response.

### Identification of predictive factors

**Figure 2:**
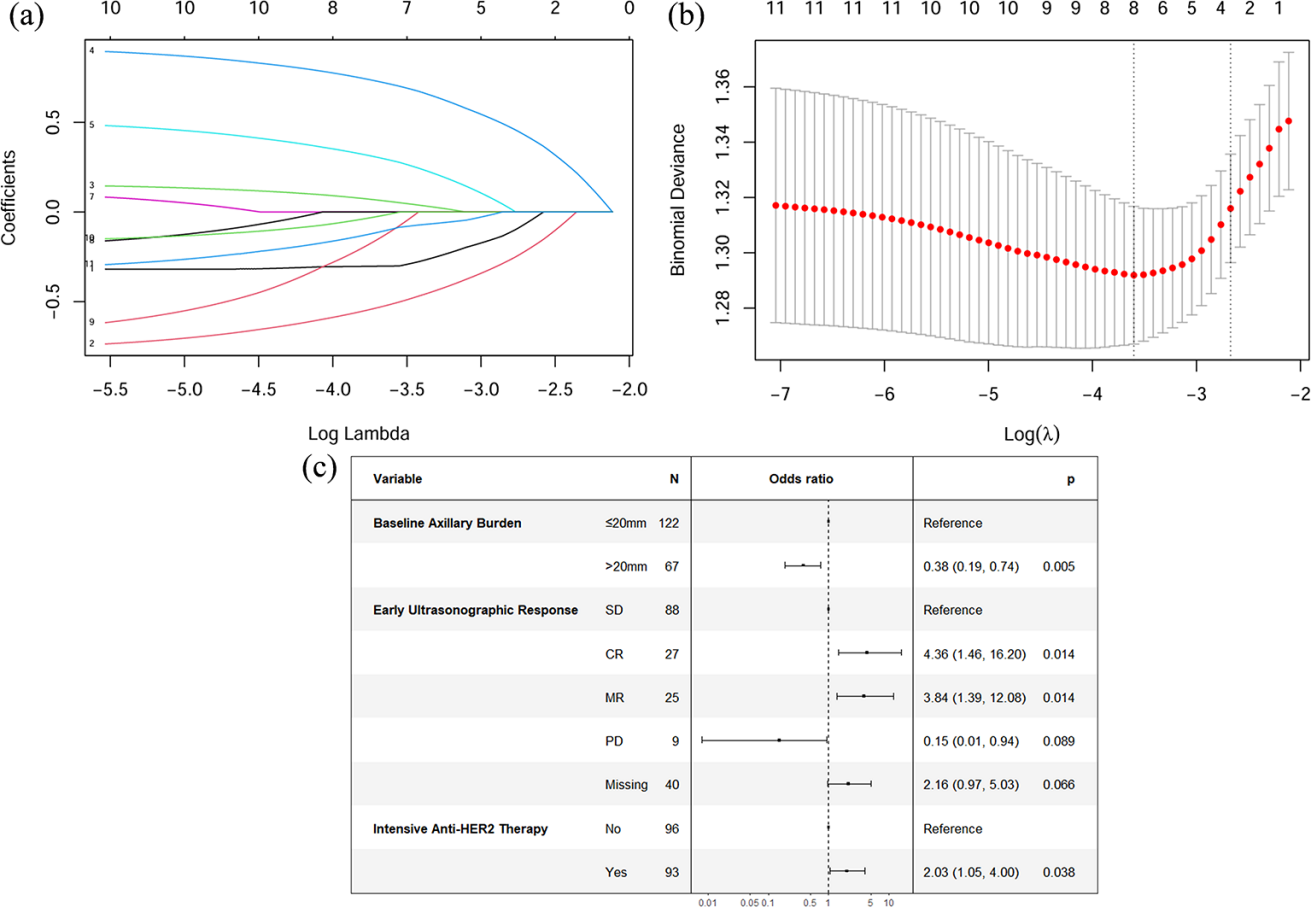
Predictor identification via LASSO and multivariable logistic regression. **(a)** A coefficient profile plot of 11 factors associated with apCR. The bottom horizontal axis represents the log lambda (λ) values of the independent variable, the top horizontal axis represents the number of variables with non-zero coefficients, and the vertical axis represents the coefficients of the independent variable. Different-colored curves represent different variables, with each curve depicting the trajectory of the coefficient variation for each independent variable. **(b)** Variable selection using 10-fold cross-validation. The dotted vertical lines indicate the optimal λ values selected by the minimum criterion (left) and the one-standard-error rule (right). The final model with eight nonzero coefficients was obtained at λ = 0.0273 (log(λ) = −3.6009). **(c)** Forest plot of the multivariable logistic regression analysis. LASSO, least absolute shrinkage and selection operator; SD, Stable Disease; CR, Complete Response; MR, Major Response; PD, Progressive Disease; HER2, human epidermal growth factor receptor 2.

To identify robust predictors of apCR while addressing potential multicollinearity, we performed feature selection using LASSO regression with 10-fold cross-validation. At the optimal λ value determined by the minimum criterion, eight predictors retained non-zero coefficients and were selected as potential variables associated with apCR (**Figures 2a, b**). These included Ki-67 index, cT stage, cN stage, clinical stage, baseline axillary burden, early ultrasonographic response, intensive anti-HER2 therapy, and number of NAT cycles. Although bpCR is a well-established predictor of apCR, it was not included in this analysis. This decision was based on the fact that techniques for preoperative bpCR assessment (e.g., minimally invasive biopsy) are not yet widely implemented in routine clinical practice (25). By focusing exclusively on readily available preoperative parameters, our model ensures broader applicability across diverse clinical settings prior to surgical intervention. Subsequent multivariable logistic regression analysis identified three independent predictors: a larger baseline axillary burden (>20 mm) was negatively associated with apCR (OR = 0.38, 95% CI: 0.19-0.75; P = 0.005), whereas an early ultrasonographic response (CR: OR = 4.36, 95% CI: 1.46-16.20; MR: OR = 3.84, 95% CI: 1.39-12.08) and intensive anti-HER2 therapy (OR = 2.03, 95% CI: 1.05-4.00) were positive predictors, with all p values < 0.05. These results are summarized in the forest plot presented in **Figure 2c**.

### Prediction model development and validation

**Figure 3:**
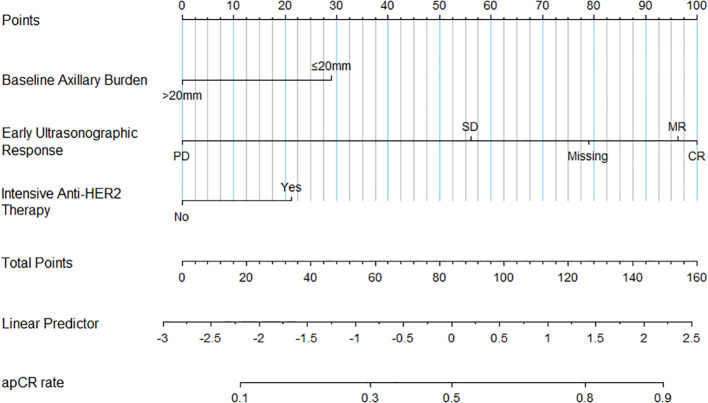
Nomogram for predicting the probability of apCR. SD, Stable Disease; CR, Complete Response; MR, Major Response; PD, Progressive Disease; HER2, human epidermal growth factor receptor 2; apCR, axillary pathological complete response. Usage Example: For a patient with a baseline axillary burden of 15 mm (≤20 mm, 28 points), an early ultrasonographic response of Major Response (MR, 96 points), and receipt of intensive anti-HER2 therapy (Yes, 22 points), the total points sum to 146. This corresponds to a predicted probability of apCR of approximately 88%.

**Figure 4:**
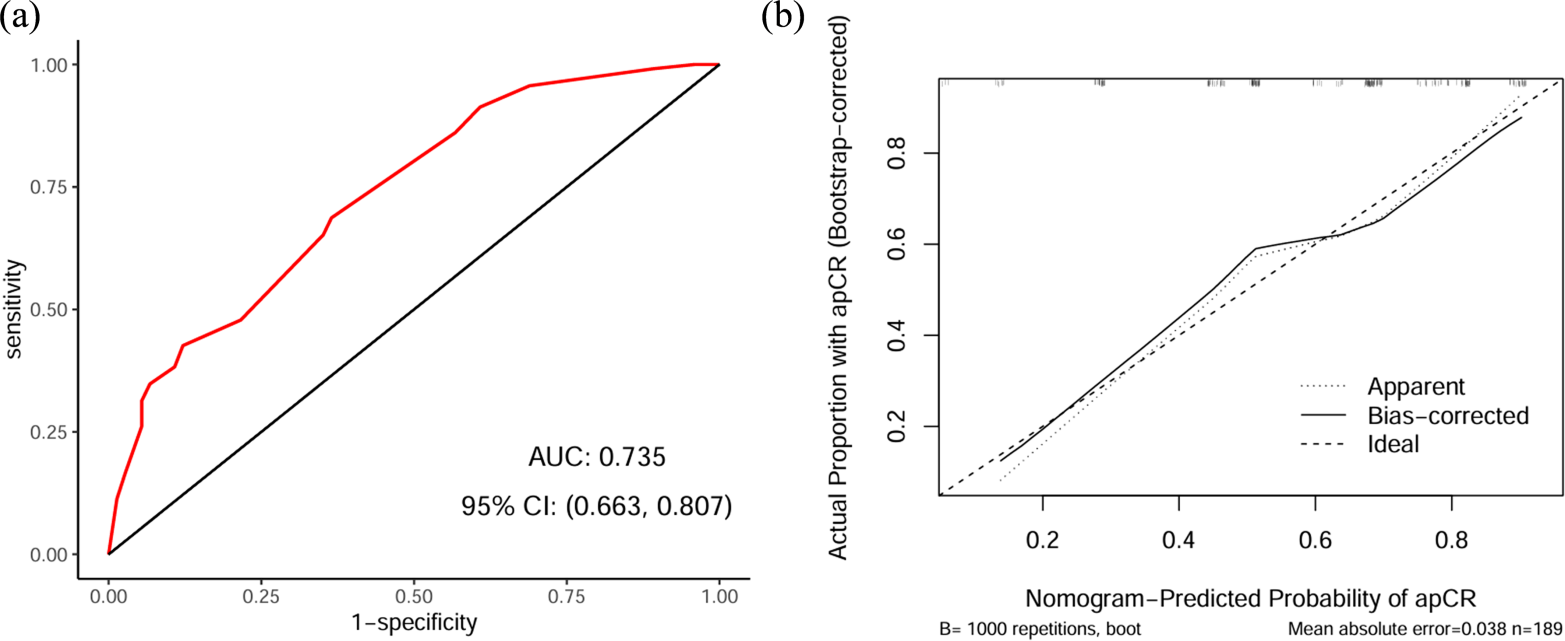
ROC curve and calibration curve of the prediction model. **(a)** ROC curve for quantifying the discriminative capacity of the model. **(b)** Calibration curve for revealing the prediction accuracy between the actual probability and the predicted probability. ROC, receiver operating characteristic; AUC, area under the receiver operating characteristic curve; CI, confidence interval; apCR, axillary pathological complete response.

**Figure 5:**
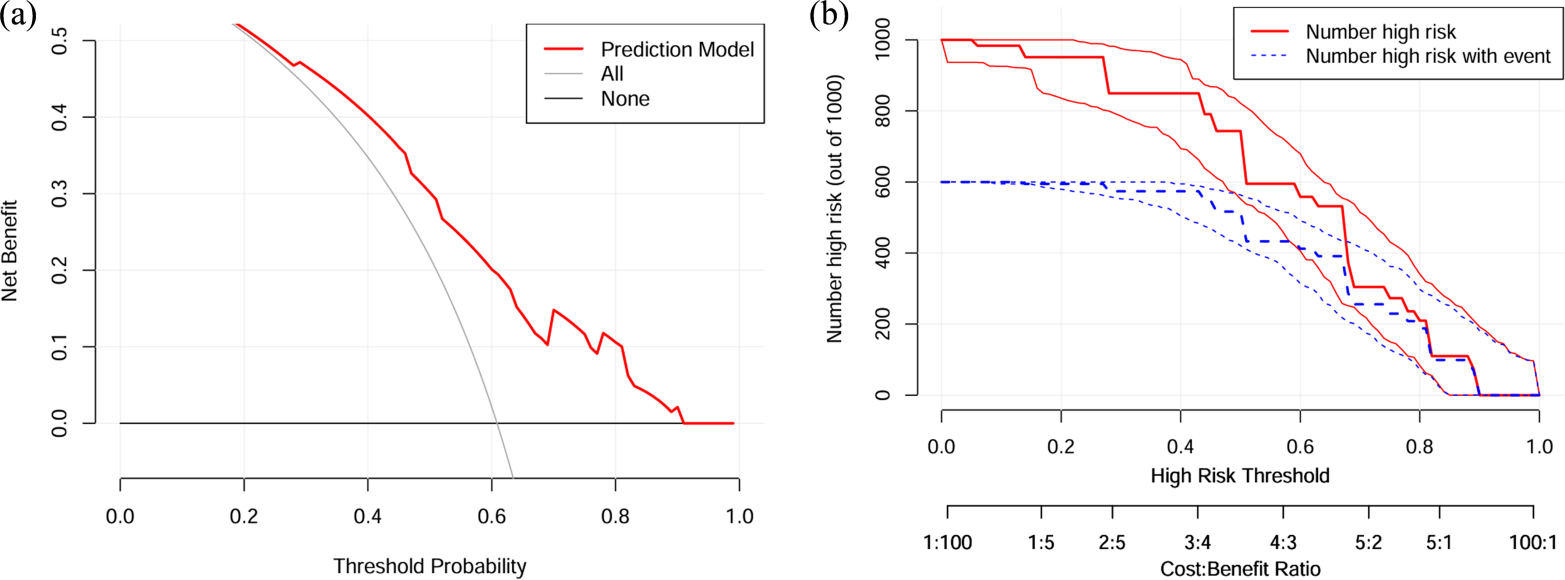
DCA and CIC for validating the clinical utility of the model. **(a)** The predictive model showed net benefit in threshold ranges of 18%-92%. **(b)** The CIC showed at threshold probabilities > 0.67, the number of high-risk individuals identified by the model closely aligned with the number of true positive cases. DCA, decision curve analysis; CIC, clinical impact curve.

A nomogram was developed by integrating the three independent predictive factors identified in the multivariate analysis. The relative contributions of these factors to the prediction of apCR were visually quantified through scaled point allocations (**Figure 3**). The discriminative performance of the nomogram was evaluated using ROC analysis, which demonstrated robust predictive performance with an AUC of 0.735 (95% CI: 0.663-0.807) (**Figure 4a**). Internal validation with 1000 bootstrap replicates yielded a calibrated AUC of 0.708 (95% CI: 0.673-0.825), supporting the model’s generalizability. The calibration plot revealed good prediction accuracy between the actual probability and the predicted probability (**Figure 4b**). DCA confirmed the clinical utility of the model, demonstrating a superior net benefit across a wide range of threshold probabilities (18%–92%) compared with the “treat all” or “treat none” strategies (**Figure 5a**). Furthermore, the CIC indicated that beyond a threshold probability of 0.67, the number of high-risk patients identified by the model closely matched the number of true positives, highlighting its accuracy in identifying individuals likely to benefit from treatment intervention (**Figure 5b**).

### Survival analysis

**Figure 6:**
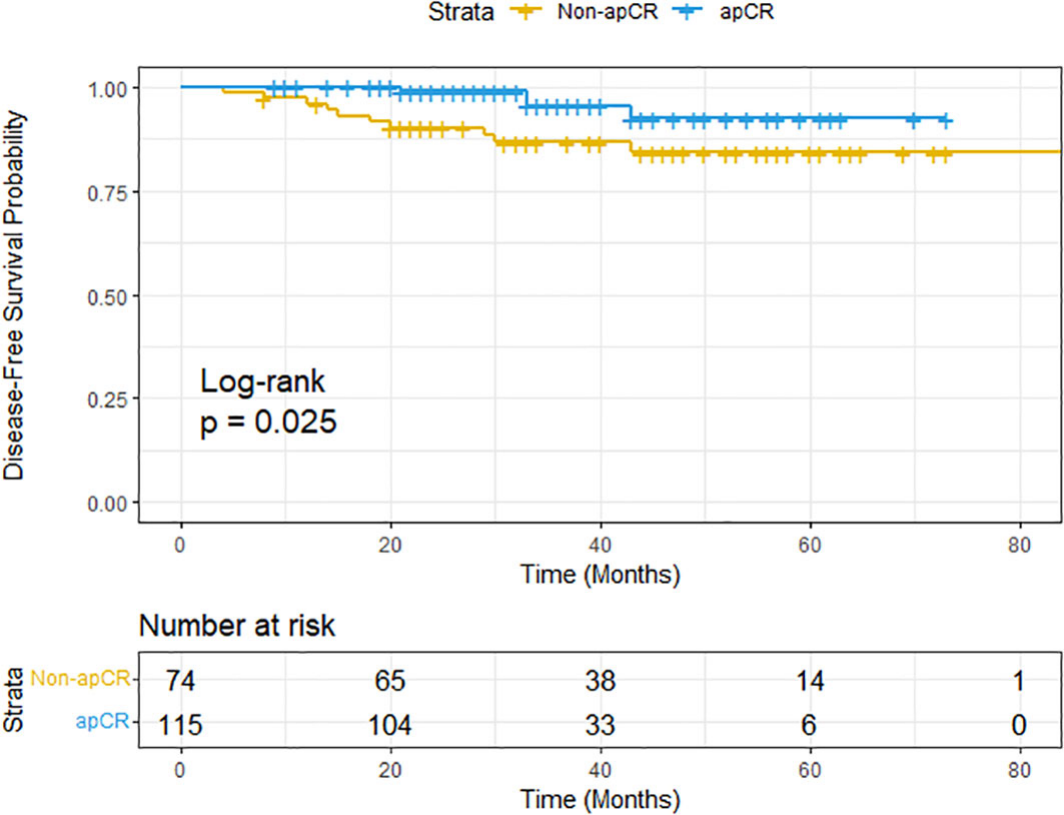
Kaplan-Meier curves comparing DFS between the apCR and non-apCR groups. DFS, disease-free survival; apCR, axillary pathological complete response.

The median follow-up for the entire cohort was 33 months. Specifically, the median follow-up was 32 months for the apCR group and 40 months for the non-apCR group. Survival analysis was performed as an exploratory analysis to evaluate the prognostic significance of apCR. The Kaplan-Meier curve demonstrated significantly superior DFS in patients achieving apCR compared to non-apCR patients (log-rank test, P = 0.025) (**Figure 6**). The apCR group consistently exhibited higher DFS rates at all landmark timepoints analyzed (**Table 2**). The 3-year DFS rate was 95.4% in the apCR group versus 86.6% in the non-apCR group. The median DFS was not reached in either group due to the favorable prognosis of the cohort. Univariable Cox proportional hazards analysis confirmed apCR as a significant prognostic factor, with patients achieving apCR experiencing a 71% reduction in the risk of recurrence or death (HR = 0.29, 95% CI: 0.09-0.92, P = 0.036).

**Table 2:** DFS rates at landmark timepoints.

Group	12-Month DFS (95% CI)	24-Month DFS (95% CI)	36-Month DFS (95% CI)	60-Month DFS (95% CI)
apCR	100% (100-100)	99.0% (97.0-100)	95.4% (90.4-100)	92.5% (85.1-100)
Non-apCR	95.9% (91.5-100)	90.3% (83.7-97.4)	86.6% (78.7-95.3)	84.3% (75.5-94.0)

DFS, disease-free survival; CI, confidence interval; apCR, axillary pathological complete response.

## Discussion

In this retrospective cohort study, we developed and internally validated a preoperative nomogram to predict apCR in HER2-positive BC. The model incorporates three readily available clinical variables: baseline axillary burden, early ultrasonographic response, and intensive anti-HER2 therapy. It demonstrated robust discriminative ability (AUC = 0.735), satisfactory calibration, and favorable clinical utility, as evidenced by DCA. Furthermore, we confirmed the significant prognostic value of apCR within our cohort, with patients achieving apCR showing markedly superior DFS. Our findings not only provide a practical predictive tool but also validate apCR as a critical endpoint, offering a comprehensive rationale for employing this model to guide personalized axillary surgical management.

The predictors integrated into our nomogram are biologically plausible and align with clinical reasoning. The early ultrasonographic response after two cycles of NAT emerged as a powerful independent predictor. This finding underscores the value of early and dynamic monitoring of treatment response. Ultrasound provides an accessible and effective means to monitor anatomical changes in axillary lymph nodes, serving as an early *in vivo* indicator of tumor sensitivity (26, 27). Consequently, a major or complete ultrasonographic response provides surgeons with valuable early information that may support the consideration of less invasive axillary procedures. Conversely, the inverse association between a larger baseline axillary burden and the likelihood of apCR extends beyond a simple anatomical measurement. This relationship may reflect tumors characterized by higher metabolic activity and inherent biological aggressiveness, which are consequently less susceptible to systemic therapy (28). Molecular evidence further substantiates this understanding, particularly through the study of minimal residual disease. The detection of circulating tumor DNA (ctDNA) after NAT, a sensitive marker of residual disease, has been strongly correlated with an increased risk of substantial residual nodal burden (29). This link implies that a substantial initial axillary tumor load frequently harbors treatment-resistant clones capable of evading systemic therapy, thereby underscoring the prognostic value of baseline axillary assessment. From a clinical standpoint, this rationale directly supports the use of precise surgical techniques such as TAD in patients with high axillary burden, ensuring the retrieval of the biopsy-proven metastatic node for accurate staging and subsequent therapy guidance (30).

Furthermore, our results strongly support the role of treatment intensity as a key determinant of apCR. Patients receiving intensive anti-HER2 therapy had a significantly higher probability of achieving apCR. This is consistent with the known synergistic mechanism of pertuzumab and trastuzumab or other tyrosine kinase inhibitors like pyrotinib in providing a more comprehensive blockade of the HER2 signaling pathway (31, 32). This finding confirms the efficacy of dual-target therapy in our real-world cohort and, crucially, identifies it as a modifiable strategy for increasing apCR rates. By incorporating this variable, our model captures a key therapeutic factor that directly influences surgical options.

The significant association between apCR and improved DFS (HR = 0.29, P = 0.036) in our cohort establishes apCR as a pivotal prognostic endpoint in HER2-positive BC, directly linked to superior long-term outcomes. This finding provides the fundamental oncologic rationale for our study: accurately identifying apCR preoperatively is of paramount importance because it demarcates a patient subgroup with a highly favorable prognosis. The strong prognostic value of apCR reinforces the biological and clinical safety of de-escalating axillary surgery in patients who achieve this endpoint.

Extensive evidence demonstrates a strong association between bpCR and axillary nodal status following NAT in HER2-positive BC patients (33, 34). Our findings further corroborate this relationship: a strong association between bpCR and apCR was observed in our cohort (73.04% vs. 29.73%, P < 0.001). When we experimentally incorporated postoperative bpCR status into the model, it yielded an AUC of 0.80, confirming its position as a powerful predictor of apCR. However, this enhanced performance comes at the cost of clinical utility. Techniques for preoperative bpCR assessment, such as vacuum-assisted biopsy, are not yet standardized or widely implemented in routine clinical practice. In current clinical practice, the definitive assessment of bpCR relies on histopathological examination of the resected breast specimen. Consequently, any model dependent on this parameter is confined to a retrospective role and cannot provide reference for surgical planning. Compared to existing models that often incorporate postoperative parameters like bpCR for predicting nodal status (11, 12), our nomogram distinguishes itself by relying exclusively on preoperative variables. This focus enhances its utility at the critical preoperative decision-making juncture, offering a practical tool for surgical planning specifically axillary de-escalation rather than retrospective assessment.

Despite the clinical utility and potential of our study, several limitations warrant consideration. First, the most significant limitation of this study stems from its retrospective, single-center design with a moderate sample size. This design inherently carries risks of selection bias and limits the generalizability of our findings. Although robust internal validation via bootstrapping was performed, the predictive performance and clinical applicability of this nomogram must be prospectively validated in larger, multi-institutional cohorts before it can be integrated into routine clinical decision-making. Second, the definition of early ultrasonographic response, while clinically practical and based on RECIST principles, possesses an inherent subjective component. Variability in imaging acquisition and interpretation across different institutions could affect the reproducibility of this predictor. Future studies incorporating more quantitative imaging biomarkers or standardized radiomic features may help to mitigate this limitation. Third, despite adjusting for key clinicopathological and treatment variables in our multivariable model, residual confounding inherent to observational studies cannot be ruled out. Unmeasured or imprecisely measured factors, such as detailed patient comorbidities, exact treatment adherence, or subtle variations in surgical technique or pathological assessment, may have influenced both the likelihood of achieving apCR and the risk of recurrence. Forth, the use of median imputation for missing baseline axillary burden data, while reasonable for this small proportion of cases, may introduce some uncertainty into the model estimates. In addition, it is important to note that the prognostic association between apCR and improved DFS, while statistically significant in our cohort, should be interpreted as preliminary. The wide confidence interval (95% CI: 0.09–0.92) around the HR reflects the limited number of DFS events, which constrains the precision of our estimate. This finding underscores the biological relevance of apCR but requires confirmation in larger studies with longer follow-up and sufficient event rates. Finally, despite our efforts to ensure complete follow-up, some patients were lost to further observation. To mitigate potential overestimation of survival rates, we conservatively censored these patients at an earlier truncation date. While this method provides a more realistic survival estimate, it cannot fully eliminate the possibility of selection bias. Future prospective, multicenter, large-scale studies are warranted to validate our findings.

## Conclusions

In conclusion, our study developed and validated a dedicated preoperative nomogram for predicting apCR in patients with HER2-positive BC. This model provides a practical and reliable tool to guide the selection of optimal candidates for axillary de-escalation strategies by integrating three readily available clinical variables: baseline axillary burden, early ultrasonographic response, and intensive anti-HER2 therapy. The validated prognostic significance of apCR in our cohort underscores its role as a pivotal trial and clinical endpoint. While external validation in prospective, multi-center studies is an indispensable next step, this nomogram represents a practical preliminary tool that could assist in preoperative surgical planning by identifying patients most likely to achieve apCR.

## Data Availability

The original contributions presented in the study are included in the article/supplementary material. Further inquiries can be directed to the corresponding author.
